# MicroRNAs miR-203-3p, miR-664-3p and miR-708-5p are associated with median strain lifespan in mice

**DOI:** 10.1038/srep44620

**Published:** 2017-03-17

**Authors:** Benjamin P. Lee, Ivana Burić, Anupriya George-Pandeth, Kevin Flurkey, David E. Harrison, Rong Yuan, Luanne L. Peters, George A. Kuchel, David Melzer, Lorna W. Harries

**Affiliations:** 1 RNA-mediated mechanisms of Disease, Institute of Biomedical and Clinical Sciences, University of Exeter Medical School, University of Exeter, Devon, UK; 2 The Jackson Laboratory Nathan Shock Center of Excellence in the Basic Biology of Aging, Bar Harbor, Maine, USA; 3 UConn Centre on Aging, University of Connecticut Health Centre, Farmington, Connecticut, USA; 4 Epidemiology and Public Health, Institute of Biomedical and Clinical Sciences, University of Exeter Medical School, University of Exeter, Devon, UK

## Abstract

MicroRNAs (miRNAs) are small non-coding RNA species that have been shown to have roles in multiple processes that occur in higher eukaryotes. They act by binding to specific sequences in the 3’ untranslated region of their target genes and causing the transcripts to be degraded by the RNA-induced silencing complex (RISC). MicroRNAs have previously been reported to demonstrate altered expression in several aging phenotypes such as cellular senescence and age itself. Here, we have measured the expression levels of 521 small regulatory microRNAs (miRNAs) in spleen tissue from young and old animals of 6 mouse strains with different median strain lifespans by quantitative real-time PCR. Expression levels of 3 microRNAs were robustly associated with strain lifespan, after correction for multiple statistical testing (miR-203-3p [β-coefficient = −0.6447, *p* = 4.8 × 10^−11^], miR-664-3p [β-coefficient = 0.5552, *p* = 5.1 × 10^−8^] and miR-708-5p [β-coefficient = 0.4986, *p* = 1.6 × 10^−6^]). Pathway analysis of binding sites for these three microRNAs revealed enrichment of target genes involved in key aging and longevity pathways including mTOR, FOXO and MAPK, most of which also demonstrated associations with longevity. Our results suggests that miR-203-3p, miR-664-3p and miR-708-5p may be implicated in pathways determining lifespan in mammals.

Although lifestyle and environmental factors are the major influences on lifespan, inherited factors remain important, with approximately 25% of the variation in lifespan attributable to genetics^1^,^2^. This is reflected in the observation that children of longer-lived parents have lower levels of age-related disease, lower all-cause mortality and greater life expectancy than those with shorter-lived parents^3^,^4^. In addition to the contribution of ‘conventional’ genetics, there is increasing evidence that epigenetic factors such as DNA methylation, histone modifications, and fine tuning of gene expression by small non-coding RNA regulators such as microRNAs (miRNAs) may also contribute significantly to aging and longevity^5^,^6^.

MicroRNAs (miRNAs) are short, non-coding RNAs that regulate mRNA expression^7^. Targets are recognized by virtue of sequence complementarity between specific sequences in the 3’ untranslated region (3’ UTR) of mRNA transcripts. Once bound, miRNAs act to either repress translation of the mRNA or target it for degradation. A single miRNA can target multiple mRNAs and many mRNAs have multiple miRNA binding sites in their 3’ UTR^8^. In this manner, miRNAs have the capacity to regulate complex networks such as those implicated in aging and longevity^9^. Several of the ‘hallmarks’ of aging^10^ including cellular senescence and genomic instability have been shown to be associated with multiple miRNAs^11^. Moreover, in several cases, individual miRNAs (or families of miRNAs) are associated with more than one of these processes^11^. Nevertheless, while several studies have implicated miRNAs in prediction of lifespan in *C. elegans*^12^,^13^,^14^, less is known about their potential role in mammalian lifespan. Identification of determinants of longevity is a key aim in identifying biomarkers of healthy aging.

Inbred strains of mice, with very well defined phenotypic characteristics and fully characterized genetics have proved a useful tool in understanding complex phenotypes such as aging^15^,^16^,^17^. In the present study, we assessed the potential role of miRNAs in longevity using spleen tissue from 6 inbred strains of mice of different median strain lifespans^15^,^16^,^17^. These mice have median strain lifespans ranging from 623 days to 1005 days and as a result we have previously used them for our studies of the factors influencing lifespan^17^,^18^. We carried out a high-throughput screen of 521 miRNAs in the young animals of the 2 strains at the extremes of the lifespan range, to identify candidate miRNAs associated with longer lifespan. We then tested for associations between median strain lifespan and the expression of the emerging miRNAs in young and old mice of all 6 strains, to determine whether these were robust associations. We found that 3 miRNAs, miR-203-3p, miR-664-3p and miR-708-5p, were all associated with lifespan in these mice. Subsequent bioinformatic analyses of pathways predicted to be targeted by these miRNAs included several that are known to be involved in determining lifespan e.g. FoxO^19^, mTOR^20^ and stem cell pluripotency pathways. Furthermore, genes predicted to be targeted by these miRNAs also show evidence of associations with median strain longevity. Our results suggest that differential regulation of key aging and longevity pathways by miRNAs may underpin some of the phenotypic variation in lifespan in mammals.

## Results

### High-throughput MicroRNA Arrays

We carried out a near-global, high throughput screen of expression of 521 miRNAs in spleen samples of young animals culled at 6 months of age from the 2 mouse strains from our collection showing the most marked divergence in lifespan (A/J; 623 days and WSB/EiJ; 1005 days) by qRT-PCR using TaqMan^®^ MicroRNA Array cards. 279 miRNAs were found to be expressed above the limit of detection and of these, 5 (miR-297b-5p, miR-708-5p, miR-224-5p, miR-203-3p and miR-327) were shown to be differentially expressed between average-lived and long-lived strains after correction for multiple testing (significance cutoff: *p* < 0.0002). Five additional miRNAs (miR-664-3p, miR-592-5p, miR-484, miR-687 and miR-192-5p) showed expression differences which were close to significance (significance cut-off: *p* < 0.002). The results for these 10 miRNAs are summarized in Table S1. See Supplementary Table S1 for results of the full analysis.

### Targeted microRNA Expression

We then measured the expression levels of the 10 miRNAs demonstrating significant or near significant associations with median strain lifespan in spleen samples from both young and old animals of all 6 mouse strains. We found that 3 miRNAs; miR-203-3p, miR-664-3p and miR-708-5p were associated with median strain lifespan (Supplementary Table S2). These 3 miRNAs were also associated with strain median lifespan in a replication sub analysis excluding all animals included in the discovery analysis (Supplementary Table S3). Analysis of expression in relation to age of the animals revealed that miR-203-3p was not significantly associated with age whereas both miR664-3p and miR-708-5p were positively associated (see Supplementary Table S4). This finding led us to perform an analysis to detect interactions between miRNA expression, age, and median strain lifespan, results of which are given in Supplementary Table S5.

**Figure 1:**
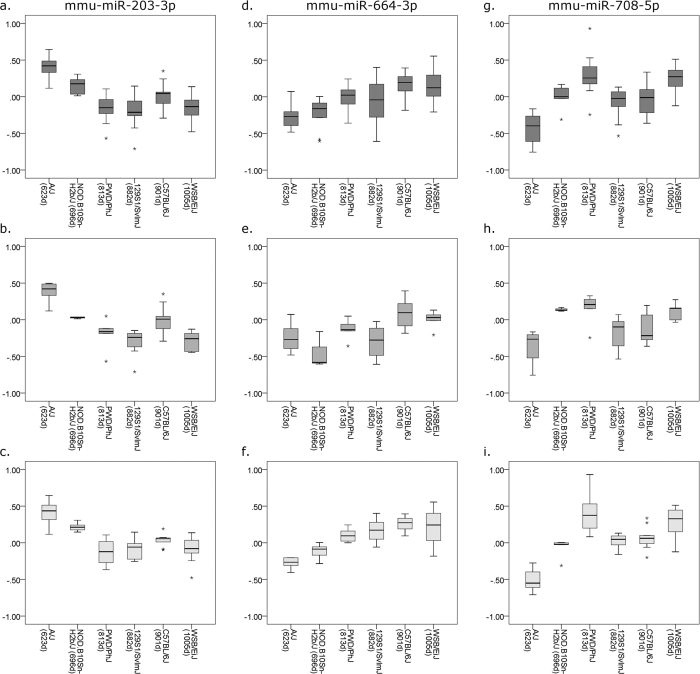
MicroRNA expression against lifespan as measured in targeted assessment of all available mouse strains. Box-and-whisker plots of relative microRNA expression for the 3 microRNAs found to be significantly associated with strain lifespan in the targeted assessment. Strains and median lifespan in days are given on the x-axis, while the y-axis shows mean log-transformed relative expression. Dark grey boxes show data for all mice analyzed, mid-grey boxes show data for young mice only and light grey boxes show data for old mice only. (**a**,**b** and **c**) expression data for miR-203-3p; (**d**,**e** and **f**) miR-664-3p; (**g**,**h** and **i**) miR-708-5p.

**Figure 2:**
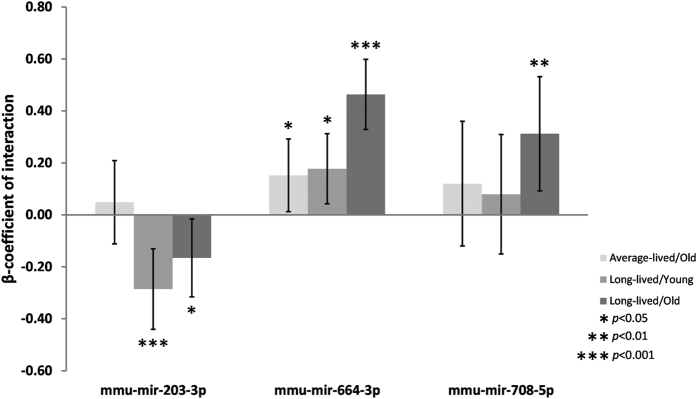
Longevity: Age interactions for microRNAs significantly associated with strain lifespan. This graph shows the relative expression changes in all mouse strains, categorized based on whether the median individual strain lifespan was above or below the median lifespan calculated across all strains, with ‘Average-lived’ being <847.5 days and ‘Long-lived’ >847.5 days. Young mice are 6 months and old mice are 20–22 months old. All changes are shown in relation to the young animals of the average-lived strains. Average-lived/old mice are shown in light grey, long-lived/young in mid-grey and long-lived/old animals in dark grey. Error bars denote the 95% confidence intervals and statistical significance is indicated by stars, where: *p < 0.05, **p < 0.01 and ***p < 0.001.

MicroRNA miR-203-3p showed significantly reduced expression in both young and old animals of strains of longer lifespan when considered separately, as well as in the analysis of old and young animals of different median strain lifespans combined, after correction for multiple testing (β-coefficients = −0.64, −0.67 and −0.67; *p* = 4.78 × 10^−11^, 3.60 × 10^−6^ and 4.74 × 10^−7^ for all, young and old analyses respectively, see Supplementary Table S2 and Fig. 1a–c). Interaction analysis revealed no significant difference between young and old animals of average-lifespan strains (β-coefficient = −0.05; SE = 0.08; *p* = 0.55, see Supplementary Table S5 and Fig. 2). However, significant expression differences were seen between strains of average lifespan and long lifespan, with the most marked differences occurring in the young animals of long-lived strains (β-coefficient = −0.29; SE = 0.08; *p* = 0.004 compared with β-coefficient = −0.17; SE = 0.08; *p* = 0.03 in the old animals of long-lived strains, see Supplementary Table S5 and Fig. 2).

Conversely, miR-664-3p demonstrated increased expression in strains of longer lifespan in both old and young animals, after correction for multiple testing (β-coefficient = 0.56, *p* = 5.12 × 10^−8^, see Supplementary Table S2 and Fig. 1d). When expression was analyzed in young animals only, a trend was observed but this did not meet multiple testing criteria (β-coefficient = 0.42, *p* = 0.008, see Supplementary Table S2 and Fig. 1e) while in the analysis of old animals only, a significant association with lifespan was seen (β-coefficient = 0.75, *p* = 3.93 × 10^−9^, see Supplementary Table S2 and Fig. 1f). Analysis of strain lifespan and age interactions for miR-664-3p showed significant differences in expression between young and old animals of average lifespan (β-coefficient = 0.15; *p* = 0.04, see Supplementary Table S5 and Fig. 2). Significant differences were also apparent when comparing expression of miR-664-3p between strains of average lifespan and long lifespan, although the effect was much more marked in the old animals of long-lived strains (β-coefficient = 0.18; *p* = 0.01 in young long-lived animals compared with β-coefficient = 0.46; *p* = 6.59 × 10^−10^, see Supplementary Table S5 and Fig. 2).

MicroRNA miR-708-5p also showed increased expression in strains of longer lifespan in the combined analysis of old and young animals, after correction for multiple testing (β-coefficient = 0.50; *p* = 1.61 × 10^−6^, see Supplementary Table S2 and Fig. 1g). Again, when expression was analyzed in young animals only, a trend was observed that did not meet multiple testing criteria (β-coefficient = 0.36; *p* = 0.02, see Supplementary Table S2 and Fig. 1h) while the analysis of old animals only showed a significant association with lifespan (β-coefficient = 0.64, *p* = 2.70 × 10^−6^, see Supplementary Table S2 and Fig. 1i). Interaction analysis for miR-708-5p showed no significant difference in expression between young and old animals of average lifespan (β-coefficient = 0.12; *p* = 0.33, see Supplementary Table S5 and Fig. 2). Significant differences were observed between average-lived and long-lived strains, but these were only present in the old animals (β-coefficient = 0.08; *p* = 0.49 in young long-lived animals compared with β-coefficient = 0.31; *p* = 0.007 in the old animals of long-lived strains, see Supplementary Table S5 and Fig. 2).

### Pathways Analysis

The lifespan effects of miR-203-3p, miR-664-3p and miR-708-5p are probably mediated by altered regulation of their target genes. We therefore used a gene set enrichment bioinformatic prediction approach specialized for miRNA targets^21^ to determine the biochemical and functional pathways that are enriched for genes targeted by the 3 microRNAs significantly associated with strain lifespan. We identified 15 pathways that were predicted to be enriched in miR-203-3p, miR-664-3p or miR-708-5p target genes, many of which are known to be associated with aging or longevity (see Table 2). Prominent pathways targeted by all 3 miRNAs include the ‘FoxO signalling pathway (mmu04068)’ and ‘mTOR signalling pathway (mmu04150)’, which contain 16 and 11 genes with predicted miR-203-3p, miR-664-3p and miR-708-5p binding sites (FDR-adjusted *p*-values = 0.02 and 0.01 respectively). Also predicted to be enriched for miR-203-3p, miR-664-3p and miR-708-5p binding sites are the ‘Pathways in cancer (mmu05200)’ pathway, the ‘MAPK signalling pathway (mmu04010), the ‘signalling pathways regulating pluripotency of stem cells (mmu04550)’ pathway and the ‘TGF-beta signalling pathway (mmu04350)’, with 33, 26, 14 and 10 genes targeted respectively (FDR-adjusted *p*-values = 0.03, 0.005, 0.05 and 0.0001 respectively). To provide empirical evidence that the genes identified to lie within these pathways also showed associations with longevity, we characterized the expression of selected target genes in relation to median strain lifespan. We identified that the 7/9 of these target genes were indeed associated with longevity in the mouse spleen samples (Table 3).

## Discussion

Even once the effects of lifestyle and environment are considered, conventional genetics cannot account for all of the variation in mammalian lifespan and other factors, such as epigenetic regulation of key genes, have also been suggested to play a role. Here we show that three miRNAs; miR-203-3p, miR-664-3p and miR-708-5p, are significantly associated with strain lifespan in mouse spleen.

The expression of miR-203-3p was negatively correlated with longer lifespan. Although effects were seen in both young and old animals of long-lived strains, the most marked effects were noted in the young animals, suggesting that modulated expression of this miRNA may be a determining factor in longevity rather than simply a consequence of advancing age. Elevated levels of miR-203-3p have previously been shown to suppress “stemness” in mouse keratinocytes with several studies finding that higher levels of miR-203-3p expression promote terminal differentiation, repress proliferation and induce senescence in human melanoma cells^22^,^23^,^24^. This microRNA has also been shown to be up-regulated in senescence in human *in vitro* models using WI-38 human diploid fibroblast cells^25^ and human melanoma cells^26^. The p63 and caveolin genes are known to be targets of miR-203-3p^27^. p63 is a member of the p53 family of transcription factors and the absence of expression of one of its isoforms, TAp63, has been shown to lead to senescence and premature aging of epidermal and dermal precursors^28^. Caveolin is thought to have a tumor-suppressor function at early stages of malignant transformation^29^, to contribute to immune senescence^30^ and the ability of aged cells to respond to oxidative stress^31^. Our finding of reduced miR-203-3p expression in long-lived mouse strains may be indicative of a phenotype in which cells have greater proliferative and adaptive capacity alongside a reduced propensity to become senescent, all of which could create favorable conditions for increased longevity. miR-203 was also one of the miRNAs demonstrated to be inversely associated with lifespan in a longitudinal study of human serum samples from the Baltimore Longitudinal Study of Aging (BLSA)^32^.

Conversely, expression of miR-664-3p showed a positive correlation with longer lifespan in our data. In contrast to miR-203-3p, the changes we noted were most evident in the old animals of the long-lived strains, suggesting that increased expression of miR-664-3p may be a later life effect on longevity. In comparison with miR-203-3p, miR-664-3p has not been extensively studied, with conflicting conclusions having been drawn by different research groups. It has been linked to both pro- and anti-proliferative action in different tumor types^33^,^34^, which complicates any attempts at prediction of putative function in terms of longevity. However, elevated hsa-miR-664 expression has been noted in human blood samples from nonagenarians and centenarians compared with samples from younger individuals^35^, indicating that in human populations, the expression of this miRNA also correlates with longevity.

MicroRNA miR-708-5p was also positively correlated with longer lifespan. Again, the changes we noted were most evident in the old animals of the long-lived strains, suggesting that increased expression of miR-708-5p may also be a later life effect on longevity. In human cells, hsa-miR-708 has been shown to have a tumor-suppressor function in several human cancer types^36^,^37^,^38^. Reduced expression of hsa-miR-708 expression has also been seen in blood taken from old individuals in comparison to young individuals^39^. In our data, elevated, rather than decreased miR-708-5p expression was found to be associated with longer lifespan. This may be partially explained if the effects on miR-708-5p expression reflect a balance between protection from malignancy and maintained proliferative capacity.

The effects of altered miRNA expression on median strain lifespan will be mediated by altered regulation of their target genes. Gene set enrichment analysis using the DIANA miRPath webtool^21^ reveals 15 pathways that are enriched for miR-203-3p, miR-664-3p and miR-708-5p target genes. The expression of the majority of the genes entriched for longevity-associated miRNA binding sites also demonstrated associations with longevity (Table 3). Although not all of these relationships were entirely straightforward in terms of the direction of effect one would predict based on expression differences of the specific miRNAs, this is to be expected, since transcripts will be targeted by many miRNAs in addition to the one tested, and several of our candidates are targeted by multiple miRNAs, often with antagonistic relationships with longevity. For example, *Zfhx3*, in the ‘pluripotency of stem cells’ pathway is targeted by both mmu-miR-203-3p and mmu-664-3p, one of which is negatively associated with lifespan and the other positively. MicroRNAs have also been previously reported to ber associated with both positive and negative associations with the expression of their target genes^40^.

Most notable amongst the pathways we found were FoxO signalling, mTOR, MAPK signaling, pathways regulating pluripotency of stem cells, TGF-beta signaling and pathways involved in cancer. FoxO is well known to be involved in the regulation of lifespan, with strong evidence that alterations in proteins in this pathway can radically increase lifespan in several model organisms as well as humans, while mTOR inhibition has also been shown to increase lifespan in several species, from yeast to mice^41^. The observation that many of the pathways implicated contain genes that are known to control shared outcomes such as apoptosis, cell cycle regulation, differentiation, proliferation, cell survival, autophagy and DNA repair adds strength to the hypothesis that miR-203-3p, miR-664-3p and miR-708-5p may have functionality in terms of longevity. Our group has previously shown that other aspects of RNA processing and regulation are important in aging and longevity in humans, in animal models and *in vitro*^17^,^42^,^43^. The results of the present study provide further evidence that post-transcriptional control of mRNA expression is a key factor in the aging process and determination of lifespan.

The use of mouse tissues from very well characterized inbred strains is a strength of our study and allows us to be precise about the genetics and phenotypes associated with each strain, and allows assessment of median strain lifespan with some confidence. Spleen is an appropriate tissue for analysis, given the known role of the immune system and inflammation as drivers of aging^44^. However, our study cannot comment on the potential tissue-specificity of the effects we have seen and may not be representative of mechanism elsewhere in the organism. We also recognize that there are both strain-specific and age-related differences in the cellular composition of the spleen. While strain differences in the cell types found in mouse spleen are apparent, the kinetics of change of cell composition with age are similar at different stages of life in separate mouse strains where this has been measured^45^. It must also be mentioned that there is a relatively large amount of inter-individual cell-type variation, in some cases more pronounced than the inter-strain variability^45^,^46^. Unfortunately, data on splenic cellular composition for the strains used in this paper are not available, however while we cannot definitively state that all of our findings are not linked to age-related cell-type changes in the splenic make-up, the associations that are present only in the young mice are far less likely to be influenced by such changes. Our use of a wide-spectrum discovery phase in a limited sample set, followed by targeted validation and replication of results in a larger inclusive cohort ensures robust results, but we recognize that for some of the mouse strains analyzed, low numbers of samples may have affected the statistical power to detect more subtle changes. The use of pathways analysis also allows a larger ‘systems’-based assessment of the effects of deregulation of modules of miRNAs in determination of longevity. Of course, it must be recognized that these results are from an *in silico* predictive algorithm and are not necessarily indicative of actual interactions *in vivo* or *in vitro*. Finally, it is possible that the effects we see may derive from differences between the strains unrelated to longevity. However, evidence suggests that there are links between both miR-664-3p and miR-203-3p and lifespan in human studies^32^,^35^, suggesting that unrelated strain differences alone probably do not account for our observations, at least for these microRNAs.

In conclusion, we present evidence that three miRNAs, miR-203-3p, miR-664-3p and miR-708-5p are robustly associated with median strain lifespan in 6 well-characterized inbred strains of mice, and that both early life (miR-203-3p) and later life (miR-664-3p and miR-708-5p) changes in their expression may modulate the expression of target genes in several very well-known aging and longevity pathways. These studies demonstrate the importance of miRNAs in determination of mammalian longevity and raise the possibility that they may have utility as biomarkers of healthy aging in the future.

## Methods

### Mouse tissue used in the study

Samples of spleen tissue were obtained from mice of six strains (A/J, NOD.B10Sn-H2^b^/J, PWD/PhJ, 129S1/SvImJ, C57BL/6 J and WSB/EiJ), selected for having variable life expectancy (see Table 4 for details of lifespan, numbers of animals used in each category and their respective characteristics). Median lifespan was measured in a longitudinal study^16^,^47^ at Jackson Laboratory Nathan Shock Center of Excellence in the Basic Biology of Aging. All tissues used in the present study were taken from male animals which were part of a cross sectional study being run at the same time, in the same mouse room as the longitudinal study mentioned above. All experiments were carried out in accordance with National Institutes of Health Laboratory Animal Care Guidelines and was approved by the Animal Care and Use Committee (ACUC) of The Jackson Laboratory. Details of mouse strains used and animal husbandry have been previously published^17^. Spleen tissue was excised immediately after death, placed into RNA-later storage solution (Sigma-Aldrich, St. Louis, MO, USA) and snap-frozen in vapor phase liquid nitrogen for storage within 5 minutes of collection.

### MicroRNA candidate transcripts for analysis

To determine which microRNA transcripts to assess for association with longevity, an initial high-throughput array analysis was performed to measure the expression of a wide spectrum of microRNAs. In an attempt to ensure the best possible chance of detecting differences with lifespan, the arrays were run using all available samples from young animals (sacrificed at 6 months old) of A/J and WSB/EiJ, the two strains at either extreme of lifespan (623 days for A/J and 1005 days for WSB/EiJ). The top 10 most significantly associated microRNAs from this analysis were followed up with targeted microRNA expression experiments in old and young animals from all 6 strains.

### RNA Extraction

Tissue samples were removed from RNA-later storage solution and placed in 1 mL TRI Reagent^®^ Solution (Thermo Fisher, Waltham, MA, USA) supplemented with the addition of 10 mM MgCl_2_ to aid recovery of microRNAs^48^. Samples were then completely homogenized for 15 mins in a bead mill (Retsch Technology GmbH, Haan, Germany). Phase separation was carried out using chloroform. Total RNA was precipitated from the aqueous phase by means of an overnight incubation at −20 °C with isopropanol. RNA pellets were then ethanol-washed twice and re-suspended in RNase-free dH2O. RNA quality and concentration was assessed by NanoDrop spectrophotometry (NanoDrop, Wilmington, DE, USA).

### High-throughput MicroRNA Arrays

#### MegaPlex Reverse Transcription

400ng of RNA per reaction was reverse transcribed using the TaqMan^®^ MicroRNA Reverse Transcription Kit and Megaplex™ RT Primers, Rodent Pool Set v3.0 (Thermo Fisher, Waltham, MA, USA) in separate reactions for Pool A and Pool B, according to the manufacturer’s instructions.

#### MicroRNA Array qRT-PCR

Expression of a wide spectrum of microRNAs was measured using Quantitative RT-PCR, performed on the ABI 7900HT platform (Thermo Fisher, Waltham, MA, USA), using both TaqMan^®^ Rodent MicroRNA A Array v2.0 and TaqMan^®^ Rodent MicroRNA Array B cards (Thermo Fisher, Waltham, MA, USA). Supplementary Table S6 lists the 521 unique microRNAs tested using this approach. Reaction mixes included 415 μl Taqman^®^ Universal PCR Master Mix II (no AmpErase^®^ UNG) (Thermo Fisher, Waltham, MA, USA), 407.5 μl dH_2_O and 7.5 μl cDNA template from Pool A or Pool B Megaplex™ reverse transcriptions as appropriate. 100 μl of reaction solution for each sample was dispensed into all chambers of an array card (again, A or B accordingly), then centrifuged twice for 1 minute at 1000 rpm to ensure distribution of solution to each well. Amplification conditions were 50 °C for 2 minutes, 94.5 °C for 10 minutes, followed by 50 cycles of 97 °C for 30 seconds and 57.9 °C for 1 minute.

### Targeted MicroRNA Expression

#### Multiplex Reverse Transcription

60 ng of RNA per reaction was reverse transcribed using the TaqMan^®^ MicroRNA Reverse Transcription Kit and RT primers provided with the TaqMan^®^ MicroRNA Assays detailed in Supplementary Table S7 (Thermo Fisher, Waltham, MA, USA). Each reaction contained 1 μl each of all the RT primers of the microRNAs to be analyzed, 1 mM dNTPs (with dTTP), 100 U MultiScribe™ Reverse Transcriptase, 1X Reverse Transcription Buffer, 7.6U RNase Inhibitor and dH_2_O to a final volume of 30 μl. The thermal profile for the reactions was 16 °C for 30 minutes, 42 °C for 30 minutes, 85 °C for 5 minutes and a final hold at 4 °C.

#### Individual microRNA qRT-PCR

MicroRNA expression was measured using Quantitative RT-PCR, performed on the ABI 7900HT platform (Thermo Fisher, Waltham, MA, USA), using the TaqMan^®^ MicroRNA Assays detailed in Supplementary Table S7 (Thermo Fisher, Waltham, MA, USA). Reactions were run in triplicate on 384-well plates, using one assay per plate containing all samples. Each reaction included 2.5 μl TaqMan^®^ Universal Master Mix II (no AmpErase^®^ UNG) and 0.25 μl TaqMan^®^ MicroRNA Assay (Thermo Fisher, Waltham, MA, USA), 0.5 μl cDNA (multiplex reverse transcribed as indicated above) and dH_2_O to a final volume of 5 μl. Amplification conditions were a single cycle of 95 °C for 10 minutes, followed by 50 cycles of 95 °C for 15 seconds and 60 °C for 1 minute.

### Interaction analysis

Analyses of interactions between mouse age and strain longevity were carried out for the three significantly associated microRNAs using data categorized based on whether the median individual strain lifespan was above or below the median lifespan calculated across all strains, with ‘average-lived’ being <847.5 days and ‘long-lived’ >847.5 days (see Table 2 for details). Interaction terms for the relationship between age and median strain longevity were included. Analyses were carried out in STATA 14 (StataCorp, College Station, TX, USA).

### Pathway analysis

Pathway analysis was carried out with DIANA-miRPath v3.0^21^, using predicted microRNA targets from the DIANA-microT-CDS v5.0 algorithm^49^ and Gene Ontology genesets derived from KEGG. The *p*-value threshold was set to 0.05 and MicroT threshold to 0.8.

### Predicted target mRNA candidates for analysis

Target genes for validation were selected based on the MiTG scores taken from the DIANA-microT-CDS v5.0 algorithm^49^. We elected to assess the two genes with the highest MiTG score from each of the three pathways with the highest numbers of genes predicted to be targeted by the microRNAs in question; ‘Pathways in cancer’ (mmu05200), ‘MAPK signalling pathway’ (mmu04010) and ‘FoxO signalling pathway’ (mmu04068). We also decided to assess the two genes with the highest MiTG score from the ‘mTOR signalling pathway’ (mmu04150) and ‘Signalling pathways regulating pluripotency of stem cells’ (mmu04550), as these were likely to be of interest in relation to lifespan. One other gene was picked (*Smad4*), as it is the only one to be present in 3 of the 5 pathways we had elected to pursue and is also present in 5 of the 15 pathways identified from DIANA-miRPath^21^.

### Predicted Target mRNA Expression

#### Reverse Transcription

200 ng of RNA per reaction was reverse transcribed using the SuperScript^®^ VILO™ cDNA Synthesis Kit (Thermo Fisher, Waltham, MA, USA) in 20 μl reactions, according to the manufacturer’s instructions. Each cDNA was then diluted with 10 μl of water to give sufficient volume to carry out the necessary qPCR reactions.

### Predicted target mRNA qRT-PCR

Predicted target mRNA expression was measured using Quantitative RT-PCR, performed on the QuantStudio 12 K Flex platform (Thermo Fisher, Waltham, MA, USA), using the TaqMan^®^ Gene Expression Assays detailed in Supplementary Table S8 (Thermo Fisher, Waltham, MA, USA). Reactions were run in triplicate on 384-well plates, using one assay per plate containing all samples. Each reaction included 2.5 μl TaqMan^®^ Universal Master Mix II (no AmpErase^®^ UNG) and 0.25 μl TaqMan^®^ Gene Expression Assay (Thermo Fisher, Waltham, MA, USA), 0.5 μl cDNA (reverse transcribed as indicated above) and dH_2_O to a final volume of 5 μl. Amplification conditions were a single cycle of 95 °C for 10 minutes, followed by 40 cycles of 95 °C for 15 seconds and 60 °C for 1 minute.

### Relative quantification

In all experiments described here, the ∆∆Ct method was used to calculate relative expression levels of the microRNAs tested^50^. Expression was assessed relative to the global mean of the 279 expressed microRNAs and normalized to the mean level of expression of each individual transcript in the shorter lifespan animals (A/J) for the high-throughput microRNA arrays. Data were log transformed to ensure normal distribution and differences in expression were tested with independent t-tests, using SPSS v22 (IBM, North Castle, NY, USA). For the targeted microRNA experiments, expression was assessed relative to the mean expression of three endogenous control small RNA species (snoRNA202, U6 snRNA and U87 snRNA) and normalized to the median level of expression for each individual transcript across all samples. Data were log10 transformed to ensure normal distribution. For the predicted target mRNA experiments, expression was assessed relative to the mean expression of two endogenous control genes (*Gusb* and *Idh3b*) and normalized to the median level of expression for each individual transcript across all samples. Data were log10 transformed to ensure normal distribution.

### Statistical approach

Associations between both miRNA and mRNA target expression and median strain lifespan were assessed using linear regression. The relationships between these parameters were assessed in both young and old animals of all 6 strains. We also assessed the relationship between median strain lifespan and miRNA expression in the animals not originally tested in the global analysis, to comprise an independent replication. Regressions were carried out using SPSS v22 (IBM, North Castle, NY, USA).

## Additional Information

**How to cite this article**: Lee, B. P. *et al*. MicroRNAs miR-203-3p, miR-664-3p and miR-708-5p are associated with median strain lifespan in mice. *Sci. Rep.* **7**, 44620; doi: 10.1038/srep44620 (2017).

**Publisher's note:** Springer Nature remains neutral with regard to jurisdictional claims in published maps and institutional affiliations.

## Supplementary Material

Supplementary Information

## Figures and Tables

**Table 1:** MicroRNAs with strongest association between expression and lifespan in spleen tissue from young mice of shortest-lived and longest-lived strains (A/J and WSB/EiJ respectively).

MicroRNA Assay ID	Mean Difference	95% CI of the difference		*p*-value
		Upper	Lower	
***mmu-miR-297b-5p***	***4.29***	***4.53***	***4.05***	***1.64* × *10*^−*11*^**
***mmu-miR-708***	***0.47***	***0.59***	***0.36***	***5.80* × *10*^*−6*^**
***mmu-miR-224***	**−*0.97***	**−*0.63***	**−*1.30***	***0.0001***
***mmu-miR-203***	**−*0.55***	**−*0.35***	**−*0.75***	***0.0002***
***rno-miR-327***	**−*3.70***	**−*2.33***	**−*5.07***	***0.0002***
mmu-miR-664	0.46	0.66	0.27	*0.0005*
mmu-miR-592	0.50	0.73	0.27	*0.0008*
mmu-miR-484	0.33	0.49	0.17	*0.001*
mmu-miR-687	5.02	7.58	2.46	*0.002*
mmu-miR-192	0.31	0.47	0.15	*0.002*

MicroRNAs significantly associated below the Bonferroni-corrected significance threshold (*p* < 0.0002) are shown in bold italics. The ten most strongly associated microRNAs followed up in the targeted analysis are shown in italics. *P*-values were determined using independent sample t-tests on log-transformed relative expression data from TaqMan^®^ MicroRNA Array cards.

**Table 2:** Pathways affected by longevity-associated microRNAs.

KEGG Pathway	*p*-value	Number of genes	Number of miRNAs
Pathways in cancer (mmu05200)	0.03	33	3
MAPK signalling pathway (mmu04010)	0.005	26	3
FoxO signalling pathway (mmu04068)	0.016	16	3
Transcriptional misregulation in cancer (mmu05202)	0.03	15	3
Signalling pathways regulating pluripotency of stem cells (mmu04550)	0.05	14	3
Thyroid hormone signalling pathway (mmu04919)	0.002	12	2
mTOR signalling pathway (mmu04150)	0.01	11	3
Long-term potentiation (mmu04720)	0.01	11	3
TGF-beta signalling pathway (mmu04350)	0.0001	10	3
Long-term depression (mmu04730)	0.002	10	2
Chronic myeloid leukaemia (mmu05220)	0.02	9	3
Amphetamine addiction (mmu05031)	0.02	8	2
Thyroid hormone synthesis (mmu04918)	0.0004	6	3
ECM-receptor interaction (mmu04512)	0.023	6	2
Glycosphingolipid biosynthesis - lacto and neolacto series (mmu00601)	1.46 × 10^−9^	4	2

DIANA-mirPath v3.0 software^21^ was used to determine pathways targeted by the microRNAs associated with strain lifespan, using predicted targets from the DIANA-microT-CDS v5.0 algorithm. Pathways are listed in order of the number of genes which are predicted to interact with these microRNAs.

**Table 3:** Association of predicted target mRNA expression and lifespan in mouse spleen tissue across 6 strains of different longevities.

Predicted Target Gene	KEGG Pathway	MicroRNA	ALL MICE			YOUNG MICE ONLY			OLD MICE ONLY		
			Beta coefficient	Std. Error	*P*-value	Beta coefficient	Std. Error	*P*-value	Beta coefficient	Std. Error	*P*-value
**Acvr2a**	Signalling pathways regulating pluripotency of stem cells (mmu04550)	mmu-miR-664-3p	−0.12	0.00	0.26	−0.38	0.00	***0.01***	0.19	0.00	0.19
**Dusp5**	MAPK signalling pathway (mmu04010)	mmu-miR-203-3p	−0.17	0.00	0.11	−0.37	0.00	***0.02***	0.03	0.00	0.84
**Fgf7**	Pathways in cancer (mmu05200)	mmu-miR-664-3p	−0.08	0.00	0.48	−0.24	0.00	0.14	0.11	0.00	0.45
	MAPK signalling pathway (mmu04010)										
**Gabarapl1**	FoxO signalling pathway (mmu04068)	mmu-miR-203-3p	−0.12	0.00	0.26	−0.48	0.00	***0.001***	0.29	0.00	***0.05***
**Mmp9**	Pathways in cancer (mmu05200)	mmu-miR-664-3p	0.45	0.00	***<0.001***	0.35	0.00	***0.02***	0.59	0.00	<***0.001***
**Pten**	FoxO signalling pathway (mmu04068)	mmu-miR-664-3p	0.18	0.00	0.09	−0.17	0.00	0.29	0.40	0.00	***0.004***
	mTOR signalling pathway (mmu04150)										
	Pathways in cancer (mmu05200)										
**Rps6ka3**	mTOR signalling pathway (mmu04150)	mmu-miR-664-3p	0.07	0.00	0.54	−0.16	0.00	0.31	0.31	0.00	***0.03***
	MAPK signalling pathway (mmu04010)										
**Smad4**	Pathways in cancer (mmu05200)	mmu-miR-664-3p	−0.36	0.00	<***0.001***	−0.38	0.00	***0.02***	−0.41	0.00	***0.004***
	FoxO signalling pathway (mmu04068)										
	Signalling pathways regulating pluripotency of stem cells (mmu04550)										
**Zfhx3**	Signalling pathways regulating pluripotency of stem cells (mmu04550)	mmu-miR-664-3p	0.03	0.00	0.80	−0.02	0.00	0.90	0.03	0.00	0.82
		mmu-miR-203-3p									

Data from mice of all ages, young mice only (6 months) and old mice only (20–22 months) are given separately. For each gene, the associated pathway is given, along with the microRNA predicted to target the transcript. mRNAs significantly associated below the significance threshold (*p* < 0.05) are shown in bold italics. *P*-values were determined from linear regression of log-transformed relative expression data.

**Table 4:** Mouse strains and characteristics.

Strain	Strain Median Lifespan (days)	Strain Maximum Age (days)	Longevity Category	n Young (6 months)	n Old (20/22 months)
**A/J**	623	785	Average lifespan	7	6
**NOD.B10Sn-H2**^**b**^**/J**	696	954	Average lifespan	3	6
**PWD/PhJ**	813	956	Average lifespan	5	6
**129S1/SvlmJ**	882	1044	Long-lived	8	8
**C57BL/6J**	901	1061	Long-lived	10	9
**WSB/EiJ**	1005	1213	Long-lived	5	10

Median lifespan and maximum age (average of longest-surviving 20% of animals) are given for each strain in the present study. All mice used were male.
