# Supplementary material for: MicroRNAs miR-203-3p, miR-664-3p and miR-708-5p are associated with median strain lifespan in mice
Source: Sci Rep. 2017 Mar 17;7:44620. doi: 10.1038/srep44620 (PMC5356331; doi:10.1038/srep44620)
Supplement: Supplementary Information [file srep44620-s1.pdf]

Supplementary Information file

MicroRNAs miR-203-3p, miR-664-3p and miR-708-5p are associated with median strain lifespan in mice.

Benjamin P. Lee, Ivana Burić, Anupriya George-Pandeth, Kevin Flurkey, David E. Harrison, Rong Yuan, Luanne L. Peters, George A. Kuchel, David Melzer and Lorna W. Harries

Supplementary Table S1. Association of MicroRNA expression and lifespan in spleen tissue from young mice of shortest-lived and longest-lived strains (A/J and WSB/EiJ respectively).

MicroRNAs significantly associated above the Bonferroni-corrected significance threshold (*p*<0.000179) are shown in bold italics. The ten most strongly associated microRNAs followed up in the targeted analysis are shown in italics. Shown in plain bold are the 6 small RNAs commonly used as endogenous controls. *P*-values were determined using independent sample t-tests on log-transformed relative expression data from TaqMan® MicroRNA Array cards.

		95% CI of the difference		
**MicroRNA Assay ID**	**Mean Difference**	**Upper**	**Lower**	***P*-value**
mmu-miR-297b-5p	***4.29***	***4.53***	***4.05***	***1.63E-11***
mmu-miR-708	***0.47***	***0.58***	***0.36***	***5.46E-06***
mmu-miR-224	***-0.97***	***-0.64***	***-1.30***	***0.0001***
mmu-miR-203	***-0.55***	***-0.35***	***-0.74***	***0.0001***
rno-miR-327	***-3.70***	***-2.33***	***-5.08***	***0.0002***
rno-miR-664	*0.46*	*0.66*	*0.27*	*0.0005*
mmu-miR-592	*0.50*	*0.73*	*0.27*	*0.0008*
mmu-miR-484	*0.33*	*0.49*	*0.17*	*0.0014*
mmu-miR-687	*5.02*	*7.58*	*2.46*	*0.0016*
mmu-miR-192	*0.31*	*0.47*	*0.15*	*0.0018*
mmu-miR-760	-0.25	-0.11	-0.38	0.003
mmu-miR-186*	0.31	0.49	0.14	0.003
mmu-miR-690	0.39	0.63	0.15	0.005
mmu-miR-31	0.43	0.69	0.16	0.005
mmu-miR-126-5p	0.26	0.42	0.09	0.006
mmu-miR-10a	-0.23	-0.08	-0.38	0.007
mmu-miR-130b*	0.36	0.59	0.12	0.007
mmu-miR-20b	0.17	0.28	0.06	0.008
mmu-miR-455*	0.25	0.42	0.08	0.009
mmu-miR-449a	-0.49	-0.15	-0.84	0.010
mmu-miR-434-3p	-0.28	-0.08	-0.49	0.011
mmu-miR-376c	-0.40	-0.10	-0.69	0.013
mmu-miR-24-2*	0.17	0.29	0.04	0.013
mmu-miR-511	-0.40	-0.09	-0.72	0.018
rno-miR-20b-5p	0.26	0.47	0.05	0.020
mmu-miR-210	0.21	0.37	0.04	0.020
mmu-miR-194	0.18	0.32	0.03	0.021
mmu-miR-411	-0.34	-0.06	-0.63	0.022
mmu-miR-875-5p	1.20	2.19	0.21	0.023
mmu-miR-340-3p	-0.27	-0.05	-0.49	0.023
mmu-miR-365	-0.17	-0.03	-0.32	0.023
mmu-miR-186	0.20	0.36	0.03	0.023
mmu-miR-539	-2.80	-0.48	-5.12	0.023
mmu-miR-140	0.12	0.23	0.01	0.030
mmu-miR-136	-0.42	-0.04	-0.79	0.033
mmu-miR-130b	0.21	0.41	0.02	0.035
mmu-miR-148b	-0.31	-0.02	-0.59	0.036
mmu-miR-31*	2.28	4.39	0.16	0.038
mmu-miR-217	-2.76	-0.15	-5.37	0.040
**snoRNA135**	**-0.11**	**0.00**	**-0.21**	**0.042**
mmu-miR-193b	0.20	0.39	0.01	0.045
mmu-miR-470*	-1.83	-0.01	-3.66	0.049
mmu-miR-877*	0.19	0.37	0.00	0.051
mmu-miR-674*	0.16	0.31	0.00	0.051

		95% CI of the difference		
**MicroRNA Assay ID**	**Mean Difference**	**Upper**	**Lower**	***P*-value**
mmu-miR-193*	0.37	0.75	-0.01	0.055
mmu-miR-700	-0.13	0.01	-0.26	0.059
mmu-miR-15a	-0.23	0.01	-0.46	0.060
mmu-miR-152	-0.19	0.01	-0.40	0.061
rno-miR-743a	-1.29	0.08	-2.66	0.062
mmu-miR-197	2.26	4.68	-0.16	0.064
mmu-miR-34b-3p	-0.22	0.02	-0.45	0.065
mmu-miR-574-3p	0.15	0.31	-0.01	0.067
mmu-miR-297a*	0.17	0.36	-0.02	0.067
mmu-miR-674	2.44	5.12	-0.24	0.070
mmu-miR-682	2.28	4.83	-0.26	0.072
mmu-miR-720	-0.13	0.02	-0.27	0.081
mmu-miR-92a	0.12	0.26	-0.02	0.089
mmu-miR-149	0.10	0.22	-0.02	0.095
mmu-miR-32	-0.24	0.05	-0.54	0.096
mmu-miR-218	-0.14	0.04	-0.32	0.103
mmu-miR-183*	-0.35	0.09	-0.79	0.106
mmu-miR-296-5p	0.22	0.49	-0.06	0.109
mmu-miR-467b*	-0.27	0.08	-0.62	0.119
mmu-miR-345-5p	-1.78	0.56	-4.12	0.119
mmu-miR-335-5p	0.14	0.32	-0.04	0.120
mmu-miR-29b	-0.13	0.04	-0.30	0.129
**MammU6**	**0.19**	**0.45**	**-0.07**	**0.130**
mmu-miR-322	-0.17	0.06	-0.40	0.132
mmu-miR-547	-1.25	0.48	-2.98	0.138
mmu-miR-467a	0.16	0.37	-0.06	0.138
mmu-miR-503	0.21	0.49	-0.08	0.141
mmu-miR-322*	-0.17	0.07	-0.40	0.142
mmu-miR-184	-0.25	0.10	-0.61	0.143
mmu-miR-195	-0.09	0.04	-0.21	0.145
mmu-miR-685	0.11	0.28	-0.05	0.147
mmu-miR-18a	-0.14	0.06	-0.33	0.152
mmu-miR-132	-0.09	0.04	-0.21	0.154
mmu-miR-135a	1.25	3.08	-0.58	0.156
mmu-miR-125b-5p	-0.09	0.04	-0.21	0.156
mmu-miR-129-3p	1.54	3.80	-0.73	0.159
mmu-miR-25	0.12	0.30	-0.06	0.163
mmu-miR-451	-0.18	0.09	-0.46	0.170
mmu-miR-744*	1.13	2.88	-0.62	0.178
mmu-miR-7a	-1.04	0.57	-2.66	0.178
mmu-miR-29a	0.07	0.18	-0.04	0.186
mmu-miR-15b*	0.14	0.36	-0.08	0.191
mmu-miR-148a	-0.22	0.13	-0.58	0.192
mmu-miR-384-5p	1.12	2.93	-0.69	0.194
mmu-let-7c	-0.15	0.09	-0.38	0.194
mmu-miR-17	0.07	0.18	-0.04	0.197
mmu-miR-211	-0.08	0.05	-0.22	0.204
mmu-miR-188-5p	0.25	0.67	-0.17	0.205
mmu-let-7b	-0.16	0.11	-0.43	0.208
mmu-miR-361	1.12	2.99	-0.76	0.211
mmu-miR-409-3p	1.00	2.69	-0.70	0.217
mmu-miR-9	1.04	2.81	-0.73	0.218

		95% CI of the difference		
**MicroRNA Assay ID**	**Mean Difference**	**Upper**	**Lower**	***P*-value**
mmu-miR-425*	-0.16	0.11	-0.42	0.222
mmu-miR-494	-0.25	0.19	-0.70	0.229
mmu-miR-130a	0.15	0.40	-0.11	0.230
**snoRNA429**	**0.46**	**1.28**	**-0.36**	**0.236**
mmu-miR-103	-0.11	0.09	-0.31	0.236
mmu-miR-29b*	-0.36	0.28	-1.01	0.237
mmu-miR-133a	0.12	0.34	-0.10	0.237
mmu-miR-125a-5p	0.10	0.27	-0.08	0.238
rno-miR-463	-0.10	0.08	-0.27	0.239
mmu-miR-29a*	0.20	0.56	-0.16	0.244
mmu-miR-29c*	-0.11	0.09	-0.30	0.245
mmu-miR-342-3p	0.09	0.26	-0.08	0.247
mmu-miR-467c	1.16	3.28	-0.97	0.249
mmu-miR-326	0.12	0.33	-0.10	0.252
mmu-miR-342-5p	0.16	0.47	-0.14	0.253
mmu-miR-30e*	0.09	0.27	-0.08	0.256
mmu-miR-542-5p	0.89	2.56	-0.78	0.257
mmu-miR-331-3p	-0.08	0.07	-0.23	0.259
mmu-miR-425	0.08	0.22	-0.07	0.268
mmu-miR-467b	0.10	0.28	-0.09	0.268
mmu-miR-30c	-0.08	0.07	-0.23	0.270
mmu-miR-128a	0.94	2.76	-0.88	0.274
mmu-miR-410	-0.80	0.78	-2.38	0.283
mmu-miR-7a*	0.09	0.27	-0.09	0.289
mmu-miR-503*	-0.15	0.16	-0.46	0.295
mmu-miR-181a-1*	0.88	2.68	-0.92	0.299
mmu-miR-335-3p	0.11	0.33	-0.12	0.303
mmu-miR-20a*	0.16	0.49	-0.17	0.306
mmu-miR-100	0.12	0.37	-0.13	0.309
mmu-miR-190b	0.38	1.19	-0.42	0.313
mmu-miR-878-3p	-1.30	1.45	-4.05	0.313
mmu-miR-30b	-0.07	0.08	-0.22	0.313
rno-miR-352	-0.93	1.04	-2.90	0.315
mmu-miR-19b	-0.08	0.09	-0.24	0.316
rno-miR-224	-1.36	1.54	-4.27	0.317
mmu-miR-143	-0.09	0.10	-0.27	0.319
mmu-miR-652	0.11	0.34	-0.12	0.319
mmu-miR-351	0.12	0.39	-0.14	0.322
mmu-miR-804	0.75	2.36	-0.87	0.322
mmu-miR-486	-0.16	0.19	-0.52	0.323
mmu-miR-93*	-0.08	0.09	-0.24	0.324
mmu-miR-125a-3p	0.16	0.51	-0.19	0.326
rno-miR-345-3p	-0.17	0.21	-0.56	0.328
mmu-miR-30a	-0.08	0.10	-0.27	0.328
mmu-miR-101b	0.07	0.22	-0.08	0.329
rno-miR-136*	-1.16	1.42	-3.74	0.337
mmu-miR-28*	-0.06	0.07	-0.19	0.339
mmu-let-7g*	0.76	2.47	-0.96	0.343
mmu-miR-466b-3-3p	0.82	2.69	-1.04	0.344
mmu-miR-21*	-0.17	0.23	-0.58	0.357
mmu-miR-98	-0.14	0.18	-0.45	0.358
rno-miR-351	0.05	0.17	-0.07	0.360

		95% CI of the difference		
**MicroRNA Assay ID**	**Mean Difference**	**Upper**	**Lower**	***P*-value**
mmu-miR-34a	0.95	3.21	-1.30	0.363
mmu-miR-324-3p	-0.11	0.16	-0.38	0.364
mmu-miR-877	-0.08	0.11	-0.27	0.364
mmu-miR-127	-0.27	0.38	-0.92	0.369
mmu-miR-362-3p	-0.25	0.36	-0.87	0.370
mmu-miR-30e	0.08	0.27	-0.11	0.371
mmu-miR-30a*	-0.05	0.07	-0.16	0.377
mmu-miR-27b	-0.09	0.12	-0.30	0.377
mmu-miR-324-5p	-0.12	0.18	-0.43	0.384
mmu-miR-680	-0.07	0.10	-0.24	0.388
rno-miR-190b	-0.74	1.11	-2.60	0.390
mmu-miR-669a	0.82	2.87	-1.24	0.393
mmu-miR-133b	0.14	0.49	-0.21	0.394
mmu-miR-126-3p	0.06	0.22	-0.10	0.398
rno-miR-7a*	-0.04	0.07	-0.16	0.412
mmu-miR-497	0.10	0.37	-0.17	0.414
mmu-miR-30d	-0.06	0.09	-0.21	0.420
mmu-let-7a*	-0.11	0.19	-0.42	0.425
mmu-miR-155	-0.06	0.11	-0.24	0.428
mmu-miR-350	0.10	0.36	-0.17	0.432
mmu-miR-218-1*	-0.77	1.36	-2.91	0.432
mmu-miR-15a*	0.08	0.31	-0.15	0.438
mmu-miR-187	-0.04	0.08	-0.16	0.441
rno-miR-30d*	-0.11	0.20	-0.42	0.441
mmu-miR-146a	-0.06	0.12	-0.24	0.446
**Y1**	**0.06**	**0.23**	**-0.11**	**0.447**
mmu-miR-135a*	-0.08	0.15	-0.31	0.448
mmu-miR-135b	0.19	0.72	-0.35	0.455
rno-miR-148b-5p	0.12	0.49	-0.24	0.455
mmu-miR-28	-0.12	0.23	-0.46	0.457
rno-miR-1	0.64	2.50	-1.22	0.457
mmu-miR-672	-0.11	0.21	-0.43	0.462
mmu-miR-532-5p	0.09	0.34	-0.17	0.462
mmu-miR-151-3p	-0.05	0.10	-0.21	0.464
mmu-miR-221	0.70	2.82	-1.43	0.478
mmu-miR-181a	0.08	0.31	-0.16	0.483
mmu-miR-532-3p	0.06	0.25	-0.13	0.486
mmu-miR-676*	0.50	2.06	-1.07	0.491
mmu-miR-378	0.11	0.47	-0.25	0.492
mmu-miR-331-5p	-0.09	0.19	-0.37	0.493
mmu-miR-27a*	-0.08	0.18	-0.35	0.497
mmu-miR-106b*	0.09	0.37	-0.20	0.504
mmu-miR-320	-0.06	0.14	-0.26	0.508
mmu-miR-339-5p	-0.06	0.15	-0.27	0.510
mmu-miR-99b*	-0.62	1.51	-2.75	0.526
mmu-miR-214	-0.07	0.18	-0.33	0.532
mmu-miR-338-3p	0.65	2.97	-1.67	0.540
mmu-miR-34c*	-0.10	0.26	-0.46	0.540
mmu-miR-214*	0.07	0.32	-0.18	0.541
mmu-miR-671-3p	0.08	0.38	-0.21	0.542
mmu-miR-21	-0.03	0.09	-0.15	0.547
mmu-let-7f	-0.04	0.10	-0.18	0.548

		95% CI of the difference		
**MicroRNA Assay ID**	**Mean Difference**	**Upper**	**Lower**	***P*-value**
rno-miR-196c	0.25	1.14	-0.65	0.550
mmu-let-7e	-0.05	0.14	-0.25	0.568
mmu-miR-124	0.76	3.65	-2.14	0.568
**snoRNA202**	**-0.05**	**0.15**	**-0.25**	**0.571**
mmu-miR-16	-0.03	0.10	-0.16	0.577
mmu-miR-200a	0.16	0.81	-0.48	0.582
mmu-miR-15b	-0.04	0.13	-0.22	0.590
mmu-miR-145	0.05	0.23	-0.14	0.591
mmu-miR-376b*	-0.24	0.73	-1.20	0.594
mmu-miR-744	-0.05	0.15	-0.25	0.601
mmu-miR-467d	0.43	2.21	-1.36	0.602
mmu-miR-99a	-0.05	0.17	-0.28	0.602
mmu-miR-23b	-0.07	0.21	-0.35	0.604
mmu-miR-141	-0.10	0.32	-0.52	0.605
mmu-miR-301a	0.06	0.32	-0.20	0.609
mmu-miR-146b	0.05	0.26	-0.16	0.615
mmu-miR-223	0.06	0.30	-0.19	0.618
mmu-miR-106b	-0.05	0.17	-0.26	0.634
mmu-miR-339-3p	-0.05	0.17	-0.26	0.643
mmu-miR-872	-0.07	0.28	-0.43	0.646
rno-miR-673	-0.14	0.51	-0.78	0.646
mmu-miR-196b	0.05	0.32	-0.21	0.652
mmu-miR-429	0.13	0.81	-0.56	0.679
mmu-miR-26b*	0.04	0.27	-0.19	0.681
mmu-miR-19a	0.04	0.24	-0.17	0.685
mmu-miR-7b	-0.12	0.56	-0.80	0.693
mmu-miR-142-5p	-0.03	0.14	-0.21	0.695
mmu-miR-340-5p	-0.04	0.16	-0.23	0.696
mmu-miR-374	0.04	0.24	-0.16	0.697
mmu-miR-676	0.04	0.27	-0.19	0.701
mmu-miR-93	-0.03	0.15	-0.22	0.704
rno-miR-204*	0.08	0.55	-0.39	0.709
mmu-miR-500	0.55	3.78	-2.68	0.710
mmu-let-7a	-0.04	0.22	-0.31	0.713
mmu-miR-101a	0.03	0.19	-0.13	0.717
mmu-let-7g	-0.03	0.13	-0.18	0.719
mmu-miR-134	0.07	0.48	-0.35	0.724
mmu-miR-99b	0.04	0.26	-0.19	0.726
mmu-miR-16*	-0.07	0.39	-0.53	0.727
mmu-miR-301b	0.04	0.30	-0.22	0.728
mmu-miR-805	-0.04	0.20	-0.27	0.739
rno-miR-339-3p	-0.03	0.19	-0.26	0.744
mmu-miR-706	-0.04	0.22	-0.30	0.747
mmu-miR-33*	-0.04	0.25	-0.34	0.753
mmu-miR-704	0.41	3.30	-2.47	0.753
mmu-miR-27b*	-0.07	0.43	-0.57	0.754
mmu-miR-204	0.02	0.18	-0.14	0.757
mmu-miR-673-5p	0.40	3.26	-2.46	0.757
**U87**	**-0.03**	**0.16**	**-0.21**	**0.759**
mmu-miR-125b*	-0.06	0.38	-0.50	0.763
mmu-miR-27a	0.03	0.23	-0.17	0.769
mmu-miR-185	0.02	0.21	-0.16	0.774

		95% CI of the difference		
**MicroRNA Assay ID**	**Mean Difference**	**Upper**	**Lower**	***P*-value**
mmu-miR-26b	0.02	0.17	-0.13	0.774
mmu-miR-1	0.06	0.58	-0.45	0.784
mmu-miR-206	-0.18	1.37	-1.73	0.799
mmu-miR-150	-0.02	0.15	-0.19	0.801
mmu-miR-193	0.04	0.44	-0.35	0.805
mmu-miR-199a-3p	-0.02	0.15	-0.19	0.807
mmu-miR-142-3p	-0.02	0.19	-0.23	0.812
mmu-miR-181c	0.04	0.45	-0.37	0.816
mmu-miR-182	-0.05	0.43	-0.52	0.819
mmu-miR-139-5p	-0.02	0.14	-0.18	0.823
mmu-miR-200b	-0.06	0.54	-0.67	0.824
mmu-miR-466d-3p	-0.03	0.25	-0.30	0.827
mmu-miR-22*	0.03	0.30	-0.24	0.828
mmu-miR-222	0.02	0.19	-0.16	0.845
mmu-miR-200c	0.03	0.38	-0.32	0.847
mmu-miR-24	-0.01	0.14	-0.17	0.852
mmu-miR-138	0.02	0.23	-0.19	0.863
mmu-miR-26a	-0.01	0.15	-0.17	0.874
mmu-miR-20a	0.01	0.13	-0.11	0.877
mmu-miR-138*	-0.04	0.65	-0.74	0.888
mmu-let-7d	-0.01	0.19	-0.22	0.896
mmu-miR-667	-0.17	2.75	-3.10	0.897
mmu-miR-328	-0.01	0.16	-0.18	0.900
mmu-miR-191	-0.01	0.19	-0.21	0.900
mmu-miR-872*	0.01	0.18	-0.16	0.907
mmu-miR-29c	-0.01	0.21	-0.24	0.909
mmu-miR-696	-0.02	0.32	-0.36	0.909
mmu-miR-376a	0.04	1.07	-0.98	0.928
mmu-miR-18a*	0.01	0.31	-0.29	0.940
mmu-miR-106a	0.01	0.19	-0.18	0.951
mmu-miR-491	-0.01	0.38	-0.39	0.974
mmu-let-7i	0.00	0.20	-0.21	0.975
mmu-miR-678	-0.03	2.67	-2.72	0.982

<u>Supplementary Table S2. Association of MicroRNA expression and lifespan in mouse spleen tissue across 6 strains of different longevities.</u>

Data from mice of all ages, young mice only (6 months) and old mice only (20-22 months) are given separately. MicroRNAs significantly associated below the Bonferroni-corrected significance threshold (p<0.005) are shown in bold italics. *P*-values were determined from linear regression of log-transformed relative expression data.

	**ALL MICE**			**YOUNG MICE ONLY**			**OLD MICE ONLY**		
	**Beta coefficient**	**Std. Error**	***P*-value**	**Beta coefficient**	**Std. Error**	***P*-value**	**Beta coefficient**	**Std. Error**	***P*-value**
**miR-192-5p**	0.16	0.00	0.14	0.12	0.00	0.49	0.20	0.00	0.20
**miR-203-3p**	***-0.64***	***0.00***	***<0.001***	***-0.67***	***0.00***	***<0.001***	***-0.67***	***0.00***	***<0.001***
**miR-224-5p**	-0.09	0.00	0.44	-0.23	0.00	0.16	0.02	0.00	0.91
**miR-297b-5p**	0.16	0.00	0.15	0.14	0.00	0.41	0.18	0.00	0.22
**miR-484**	-0.07	0.00	0.55	-0.21	0.00	0.21	0.08	0.00	0.61
**miR-592**	0.16	0.00	0.15	0.08	0.00	0.65	0.23	0.00	0.13
**miR-664-3p**	***0.56***	***0.00***	***<0.001***	0.42	0.00	0.01	***0.75***	***0.00***	***<0.001***
**miR-687**	0.20	0.00	0.11	0.17	0.00	0.39	0.23	0.00	0.18
**miR-708-5p**	***0.50***	***0.00***	***<0.001***	0.37	0.00	0.02	***0.64***	***0.00***	***<0.001***
**miR-327**	-0.21	0.00	0.06	-0.38	0.00	0.02	-0.09	0.00	0.54

Supplementary Table S3. Sub analysis of the relationship between miRNA expression and median strain longevity in spleen samples from animals not included in the initial global analysis.

Data from mice of all ages, young mice only (6 months) and old mice only (20-22 months) are given separately. MicroRNAs significantly associated below the Bonferroni-corrected significance threshold (p<0.005) are shown in bold italics. *P*-values were determined from linear regression of log-transformed relative expression data.

	ALL MICE			YOUNG MICE ONLY			OLD MICE ONLY		
	**Beta coefficient**	**Std. Error**	***P*-value**	**Beta coefficient**	**Std. Error**	***P*-value**	**Beta coefficient**	**Std. Error**	***P*-value**
**miR-192-5p**	0.08	0.00	0.50	-0.21	0.00	0.30	0.20	0.00	0.20
**miR-203-3p**	***-0.51***	***0.00***	***<0.001***	-0.10	0.00	0.64	***-0.70***	***0.00***	***<0.001***
**miR-224-5p**	0.17	0.00	0.16	***0.82***	***0.00***	***<0.001***	0.02	0.00	0.90
**miR-297b-5p**	0.11	0.00	0.38	-0.20	0.00	0.33	0.19	0.00	0.22
**miR-484**	0.12	0.00	0.30	0.31	0.00	0.12	0.08	0.00	0.61
**miR-592**	0.10	0.00	0.41	-0.38	0.00	0.06	0.22	0.00	0.15
**miR-664-3p**	***0.56***	***0.00***	***<0.001***	0.47	0.00	0.02	***0.75***	***0.00***	***<0.001***
**miR-687**	0.17	0.00	0.21	0.02	0.01	0.93	0.23	0.00	0.18
**miR-708-5p**	***0.41***	***0.00***	***<0.001***	-0.50	0.01	0.01	***0.64***	***0.00***	***<0.001***
**rno-miR-327**	-0.18	0.00	0.14	***-0.54***	***0.00***	***0.004***	-0.10	0.00	0.54

Supplementary Table S4. Association of MicroRNA expression and age in mouse spleen tissue across 6 strains of different longevities.

Data from mice of all ages, 'Average-lived' mice only (<847.5 days) and 'Long-lived' mice only (>847.5 days) are given separately. MicroRNAs significantly associated below the Bonferroni-corrected significance threshold (*p*<0.005) are shown in bold italics. *P*-values were determined from linear regression of log-transformed relative expression data.

	ALL MICE			AVERAGE-LIVED MICE ONLY			LONG-LIVED MICE ONLY		
	**Beta coefficient**	**Std. Error**	***P*-value**	**Beta coefficient**	**Std. Error**	***P*-value**	**Beta coefficient**	**Std. Error**	***P*-value**
**miR-192-5p**	0.12	0.04	0.27	0.00	0.07	0.99	0.20	0.05	0.16
**miR-203-3p**	0.18	0.06	0.11	0.09	0.10	0.63	0.30	0.05	0.03
**miR-224-5p**	-0.01	0.11	0.95	-0.14	0.17	0.44	0.09	0.13	0.55
**miR-297b-5p**	-0.09	0.08	0.41	-0.10	0.15	0.58	-0.09	0.09	0.55
**miR-484**	0.10	0.04	0.35	0.04	0.06	0.81	0.14	0.05	0.33
**miR-592**	***0.44***	***0.05***	***<0.001***	0.37	0.07	0.03	***0.57***	***0.06***	***<0.001***
**miR-664-3p**	0.04	0.41	0.77	0.12	0.41	0.58	0.02	0.62	0.90
**miR-687**	0.26	0.08	0.02	0.12	0.17	0.49	***0.50***	***0.06***	***<0.001***
**miR-708-5p**	0.03	0.09	0.77	-0.05	0.17	0.78	0.13	0.08	0.38
**rno-miR-327**	-0.08	0.12	0.46	-0.07	0.22	0.72	-0.11	0.13	0.43

Supplementary Table S5. Analyses of potential interactions between mouse strain longevity and mouse age.

Std. Error = standard error, 95% CI = 95% confidence intervals. Mouse strains were categorised for this analysis based on whether the median individual strain lifespan was above or below the median lifespan calculated across all strains, with 'Average-lived' being <847.5 days and 'Long-lived' >847.5 days. Young mice are 6 months and old mice are 20-22 months old. Statistically significant results are indicated in bold italic text.

Longevity:age interactions - microRNAs associated with lifespan					
**MicroRNA**	**Sub-category**	**β coefficient**	**Std. Error**	**95% CI**	***p*-value**
**mmu-miR-203-3p**	Average-lived/Young<br>Average-lived/Old<br>Long-lived/Young<br>Long-lived/Old	0<br>0.05<br>-0.29<br>-0.17	 0.08<br>0.08<br>0.08	 -0.11 to 0.21<br>-0.44 to -0.13<br>-0.32 to -0.02	 0.55<br>***0.0004***<br>***0.03***
**mmu-miR-664-3p**	Average-lived/Young<br>Average-lived/Old<br>Long-lived/Young<br>Long-lived/Old	0<br>0.15<br>0.18<br>0.46	 0.07<br>0.07<br>0.07	 0.01 to 0.29<br>0.04 to 0.31<br>0.33 to 0.60	 ***0.04***<br>***0.01***<br>***6.59E-10***
**mmu-miR-708-5p**	Average-lived/Young<br>Average-lived/Old<br>Long-lived/Young<br>Long-lived/Old	0<br>0.12<br>0.08<br>0.31	 0.12<br>0.12<br>0.11	 -0.12 to 0.36<br>-0.15 to 0.31<br>0.09 to 0.53	 0.33<br>0.49<br>***0.007***

Supplementary Table S6. Taqman® Low Density Array card contents.

Assay names and unique assay IDs are given for all microRNAs tested using each array layout.

Rodent A Array v2.0					
**Assay ID**	**Assay Name**	**Assay ID**	**Assay Name**	**Assay ID**	**Assay Name**
000377	mmu-let-7a	002592	mmu-miR-291a-3p	002456	mmu-miR-503
000378	mmu-let-7b	002537	mmu-miR-291b-5p	002084	mmu-miR-504
000379	mmu-let-7c	002593	mmu-miR-292-3p	001655	mmu-miR-505
002283	mmu-let-7d	001794	mmu-miR-293	002521	mmu-miR-509-3p
002406	mmu-let-7e	001056	mmu-miR-294	002520	mmu-miR-509-5p
000382	mmu-let-7f	000189	mmu-miR-295	002549	mmu-miR-511
002282	mmu-let-7g	002101	mmu-miR-296-3p	002355	mmu-miR-532-3p
002221	mmu-let-7i	000527	mmu-miR-296-5p	001518	mmu-miR-532-5p
002222	mmu-miR-1	001626	mmu-miR-297b-5p	001286	mmu-miR-539
000437	mmu-miR-100	002480	mmu-miR-297c	001310	mmu-miR-540-3p
002253	mmu-miR-101a	002598	mmu-miR-298	002561	mmu-miR-540-5p
000439	mmu-miR-103	002112	mmu-miR-29a	001284	mmu-miR-542-3p
002465	mmu-miR-105	000413	mmu-miR-29b	002563	mmu-miR-542-5p
002459	mmu-miR-106a	000587	mmu-miR-29c	002376	mmu-miR-543
000442	mmu-miR-106b	000528	mmu-miR-301a	002550	mmu-miR-544
000443	mmu-miR-107	002600	mmu-miR-301b	001312	mmu-miR-546
000387	mmu-miR-10a	000529	mmu-miR-302a	002564	mmu-miR-547
002218	mmu-miR-10b	000531	mmu-miR-302b	001535	mmu-miR-551b
002245	mmu-miR-122	002558	mmu-miR-302c	002349	mmu-miR-574-3p
001182	mmu-miR-124	000535	mmu-miR-302d	002567	mmu-miR-582-3p
002199	mmu-miR-125a-3p	000417	mmu-miR-30a	002566	mmu-miR-582-5p
002198	mmu-miR-125a-5p	000602	mmu-miR-30b	001984	mmu-miR-590-5p
002378	mmu-miR-125b-3p	000419	mmu-miR-30c	002476	mmu-miR-598
000449	mmu-miR-125b-5p	000420	mmu-miR-30d	001960	mmu-miR-615-3p
002228	mmu-miR-126-3p	002223	mmu-miR-30e	002353	mmu-miR-615-5p
000451	mmu-miR-126-5p	000185	mmu-miR-31	002352	mmu-miR-652
000452	mmu-miR-127	002109	mmu-miR-32	002239	mmu-miR-654-3p
002216	mmu-miR-128a	002277	mmu-miR-320	002522	mmu-miR-654-5p
001184	mmu-miR-129-3p	001076	mmu-miR-322	002607	mmu-miR-665
000590	mmu-miR-129-5p	002227	mmu-miR-323-3p	001952	mmu-miR-666-5p
000454	mmu-miR-130a	002509	mmu-miR-324-3p	001949	mmu-miR-667
000456	mmu-miR-130b	000539	mmu-miR-324-5p	001947	mmu-miR-668
000457	mmu-miR-132	002510	mmu-miR-325	001683	mmu-miR-669a
002246	mmu-miR-133a	000543	mmu-miR-328	002020	mmu-miR-670
002247	mmu-miR-133b	000192	mmu-miR-329	002322	mmu-miR-671-3p
001186	mmu-miR-134	002230	mmu-miR-330	002327	mmu-miR-672
000460	mmu-miR-135a	000545	mmu-miR-331-3p	002021	mmu-miR-674
002261	mmu-miR-135b	002233	mmu-miR-331-5p	001941	mmu-miR-675-3p
002511	mmu-miR-136	002185	mmu-miR-335-3p	001940	mmu-miR-675-5p
001129	mmu-miR-137	000546	mmu-miR-335-5p	001959	mmu-miR-676
002284	mmu-miR-138	002532	mmu-miR-337-3p	001660	mmu-miR-677
002546	mmu-miR-139-3p	002515	mmu-miR-337-5p	001662	mmu-miR-679
002289	mmu-miR-139-5p	002252	mmu-miR-338-3p	001664	mmu-miR-680
001187	mmu-miR-140	002533	mmu-miR-339-3p	001666	mmu-miR-682
000463	mmu-miR-141	002257	mmu-miR-339-5p	001668	mmu-miR-683
000464	mmu-miR-142-3p	002259	mmu-miR-340-3p	001669	mmu-miR-684
002248	mmu-miR-142-5p	002258	mmu-miR-340-5p	001670	mmu-miR-685
002249	mmu-miR-143	002260	mmu-miR-342-3p	001672	mmu-miR-686
002278	mmu-miR-145	002527	mmu-miR-342-5p	001674	mmu-miR-687

Rodent A Array v2.0					
Assay ID	Assay Name	Assay ID	Assay Name	Assay ID	Assay Name
000468	mmu-miR-146a	001063	mmu-miR-344	002341	mmu-miR-708
001097	mmu-miR-146b	002529	mmu-miR-345-3p	002457	mmu-miR-741
002262	mmu-miR-147	002528	mmu-miR-345-5p	002038	mmu-miR-742
000470	mmu-miR-148a	001064	mmu-miR-346	002469	mmu-miR-743a
000471	mmu-miR-148b	000426	mmu-miR-34a	002471	mmu-miR-743b-3p
000473	mmu-miR-150	002618	mmu-miR-34b-3p	002470	mmu-miR-743b-5p
001190	mmu-miR-151-3p	000428	mmu-miR-34c	002324	mmu-miR-744
000475	mmu-miR-152	002530	mmu-miR-350	002027	mmu-miR-770-3p
001191	mmu-miR-153	001067	mmu-miR-351	000268	mmu-miR-7a
000477	mmu-miR-154	000554	mmu-miR-361	002555	mmu-miR-7b
002571	mmu-miR-155	002616	mmu-miR-362-3p	002029	mmu-miR-802
000389	mmu-miR-15a	001271	mmu-miR-363	002354	mmu-miR-871
000390	mmu-miR-15b	001020	mmu-miR-365	002264	mmu-miR-872
000391	mmu-miR-16	000555	mmu-miR-367	002356	mmu-miR-873
002308	mmu-miR-17	000557	mmu-miR-369-3p	002268	mmu-miR-874
000480	mmu-miR-181a	001021	mmu-miR-369-5p	002547	mmu-miR-875-3p
000482	mmu-miR-181c	002275	mmu-miR-370	002464	mmu-miR-876-3p
002599	mmu-miR-182	000564	mmu-miR-375	002463	mmu-miR-876-5p
002269	mmu-miR-183	001069	mmu-miR-376a	002540	mmu-miR-878-5p
000485	mmu-miR-184	002452	mmu-miR-376b	002472	mmu-miR-879
002271	mmu-miR-185	002450	mmu-miR-376c	002609	mmu-miR-881
002285	mmu-miR-186	000566	mmu-miR-377	002461	mmu-miR-883a-3p
001193	mmu-miR-187	001138	mmu-miR-379	002611	mmu-miR-883a-5p
002106	mmu-miR-188-3p	001071	mmu-miR-380-3p	002565	mmu-miR-883b-3p
002320	mmu-miR-188-5p	002601	mmu-miR-380-5p	000583	mmu-miR-9
002422	mmu-miR-18a	000571	mmu-miR-381	000430	mmu-miR-92a
002466	mmu-miR-18b	000572	mmu-miR-382	001090	mmu-miR-93
000489	mmu-miR-190	001767	mmu-miR-383	000186	mmu-miR-96
002299	mmu-miR-191	002603	mmu-miR-384-3p	000577	mmu-miR-98
000491	mmu-miR-192	002602	mmu-miR-384-5p	000435	mmu-miR-99a
002250	mmu-miR-193	002332	mmu-miR-409-3p	000436	mmu-miR-99b
002467	mmu-miR-193b	002331	mmu-miR-409-5p	002064	rno-miR-1
000493	mmu-miR-194	001274	mmu-miR-410	002078	rno-miR-17-3p
000494	mmu-miR-195	001610	mmu-miR-411	002048	rno-miR-190b
002215	mmu-miR-196b	002340	mmu-miR-423-5p	002049	rno-miR-196c
000497	mmu-miR-197	001516	mmu-miR-425	001315	rno-miR-207
002304	mmu-miR-199a-3p	001077	mmu-miR-429	002052	rno-miR-20b-3p
000498	mmu-miR-199a-5p	001979	mmu-miR-431	002077	rno-miR-219-1-3p
000395	mmu-miR-19a	001028	mmu-miR-433	002390	rno-miR-219-2-3p
000396	mmu-miR-19b	002604	mmu-miR-434-3p	000599	rno-miR-224
000502	mmu-miR-200a	002581	mmu-miR-434-5p	001328	rno-miR-327
002251	mmu-miR-200b	001029	mmu-miR-448	001329	rno-miR-333
002300	mmu-miR-200c	001030	mmu-miR-449a	001331	rno-miR-336
002578	mmu-miR-201	002539	mmu-miR-449b	002059	rno-miR-339-3p
001195	mmu-miR-202-3p	002303	mmu-miR-450a-5p	001344	rno-miR-343
000507	mmu-miR-203	001962	mmu-miR-450b-5p	001332	rno-miR-344-3p
000508	mmu-miR-204	001141	mmu-miR-451	002060	rno-miR-344-5p
000509	mmu-miR-205	001032	mmu-miR-452	002061	rno-miR-345-3p
001198	mmu-miR-207	002484	mmu-miR-453	001333	rno-miR-346
000511	mmu-miR-208	002455	mmu-miR-455	001334	rno-miR-347
002290	mmu-miR-208b	001081	mmu-miR-464	001335	rno-miR-349
000580	mmu-miR-20a	002040	mmu-miR-465a-3p	002063	rno-miR-351

Rodent A Array v2.0					
Assay ID	Assay Name	Assay ID	Assay Name	Assay ID	Assay Name
001014	mmu-miR-20b	001082	mmu-miR-465a-5p	001320	rno-miR-377
000397	mmu-miR-21	002485	mmu-miR-465b-5p	001322	rno-miR-381
000512	mmu-miR-210	002516	mmu-miR-466h	001317	rno-miR-409-5p
001199	mmu-miR-211	002587	mmu-miR-467a	001343	rno-miR-421
002306	mmu-miR-214	001671	mmu-miR-467b	001345	rno-miR-450a
001200	mmu-miR-215	002517	mmu-miR-467c	002066	rno-miR-466b
002220	mmu-miR-216a	002518	mmu-miR-467d	002067	rno-miR-466c
002326	mmu-miR-216b	002568	mmu-miR-467e	001316	rno-miR-505
002556	mmu-miR-217	001085	mmu-miR-468	002051	rno-miR-532-5p
000521	mmu-miR-218	001086	mmu-miR-469	002065	rno-miR-543
000522	mmu-miR-219	002588	mmu-miR-470	002053	rno-miR-598-5p
002468	mmu-miR-220	001821	mmu-miR-484	002054	rno-miR-673
000524	mmu-miR-221	001278	mmu-miR-486	002055	rno-miR-742
002276	mmu-miR-222	001285	mmu-miR-487b	002068	rno-miR-743b
002295	mmu-miR-223	001659	mmu-miR-488	001990	rno-miR-758
002553	mmu-miR-224	001302	mmu-miR-489	002057	rno-miR-760-5p
000399	mmu-miR-23a	001037	mmu-miR-490	002069	rno-miR-871
000400	mmu-miR-23b	001630	mmu-miR-491	002070	rno-miR-878
000402	mmu-miR-24	002519	mmu-miR-493	002072	rno-miR-881
000403	mmu-miR-25	002365	mmu-miR-494	000338	ath-miR159a
000405	mmu-miR-26a	001663	mmu-miR-495	001973	Mamm U6
000407	mmu-miR-26b	001953	mmu-miR-496	001230	snoRNA135
000408	mmu-miR-27a	001346	mmu-miR-497	001232	snoRNA202
000409	mmu-miR-27b	001352	mmu-miR-499	001712	U87
000411	mmu-miR-28	002606	mmu-miR-500	001727	Y1
002591	mmu-miR-290-3p	001651	mmu-miR-501-3p		

Rodent Array B					
Assay ID	Assay Name	Assay ID	Assay Name	Assay ID	Assay Name
002478	mmu-let-7a*	002498	mmu-miR-30b*	001642	mmu-miR-707
002479	mmu-let-7c-1*	002495	mmu-miR-31*	001646	mmu-miR-711
001178	mmu-let-7d*	002506	mmu-miR-322*	001961	mmu-miR-712*
002492	mmu-let-7g*	001060	mmu-miR-325*	001648	mmu-miR-713
002507	mmu-miR-101a*	001061	mmu-miR-326	001649	mmu-miR-715
002531	mmu-miR-101b	002481	mmu-miR-327	001652	mmu-miR-717
002572	mmu-miR-10b*	002136	mmu-miR-33*	001656	mmu-miR-718
002508	mmu-miR-125b*	001062	mmu-miR-330*	001673	mmu-miR-719
002229	mmu-miR-127*	002483	mmu-miR-343	001629	mmu-miR-720
002460	mmu-miR-130b*	002584	mmu-miR-34c*	001657	mmu-miR-721
001637	mmu-miR-133a*	002043	mmu-miR-374*	002034	mmu-miR-759
002512	mmu-miR-136*	002482	mmu-miR-376a*	002028	mmu-miR-762
002554	mmu-miR-138*	002451	mmu-miR-376b*	002033	mmu-miR-763
002513	mmu-miR-141*	002523	mmu-miR-376c*	002032	mmu-miR-764-3p
002514	mmu-miR-145*	001078	mmu-miR-433*	002031	mmu-miR-764-5p
002453	mmu-miR-146b*	002525	mmu-miR-450a-3p	002044	mmu-miR-804
002570	mmu-miR-150*	002582	mmu-miR-463*	002045	mmu-miR-805
002488	mmu-miR-15a*	002586	mmu-miR-466a-3p	002542	mmu-miR-872*
002489	mmu-miR-16*	002500	mmu-miR-466b-3-3p	002548	mmu-miR-877*
002543	mmu-miR-17*	002534	mmu-miR-466d-5p	002541	mmu-miR-878-3p
002270	mmu-miR-183*	001826	mmu-miR-467a*	002473	mmu-miR-879*
002574	mmu-miR-186*	001684	mmu-miR-467b*	002475	mmu-miR-881*

Rodent Array B					
Assay ID	Assay Name	Assay ID	Assay Name	Assay ID	Assay Name
002490	mmu-miR-18a*	002569	mmu-miR-467e*	002231	mmu-miR-9*
002576	mmu-miR-191*	002589	mmu-miR-470*	002496	mmu-miR-92a*
002577	mmu-miR-193*	002560	mmu-miR-483*	001351	rno-miR-1*
002477	mmu-miR-196a*	001943	mmu-miR-485*	002074	rno-miR-125b*
001131	mmu-miR-199b*	002014	mmu-miR-488*	002075	rno-miR-135a*
002544	mmu-miR-19a*	002536	mmu-miR-503*	002058	rno-miR-148b-5p
002491	mmu-miR-20a*	002017	mmu-miR-592	002076	rno-miR-204*
002524	mmu-miR-20b*	002449	mmu-miR-673-3p	001336	rno-miR-20a*
002493	mmu-miR-21*	001956	mmu-miR-674*	002079	rno-miR-24-1*
002293	mmu-miR-214*	001958	mmu-miR-676*	002080	rno-miR-25*
002552	mmu-miR-218-1*	001675	mmu-miR-688	002082	rno-miR-29b-1*
002494	mmu-miR-24-2*	001677	mmu-miR-690	001339	rno-miR-352
002545	mmu-miR-28*	001678	mmu-miR-691	002081	rno-miR-379*
002538	mmu-miR-291b-3p	001679	mmu-miR-692	001354	rno-miR-382*
001055	mmu-miR-292-5p	002036	mmu-miR-693-3p	001353	rno-miR-489
002594	mmu-miR-293*	001681	mmu-miR-694	001323	rno-miR-664
002595	mmu-miR-294*	001627	mmu-miR-695	002056	rno-miR-743a
002454	mmu-miR-297a*	001628	mmu-miR-696	002062	rno-miR-7a*
000600	mmu-miR-299*	001631	mmu-miR-697	002073	rno-miR-99a*
002497	mmu-miR-29b*	001632	mmu-miR-698	000338	ath-miR159a
000191	mmu-miR-300	001634	mmu-miR-700	001973	Mamm U6
002613	mmu-miR-300*	001635	mmu-miR-701	001230	snoRNA135
002615	mmu-miR-302a*	001636	mmu-miR-702	001232	snoRNA202
001307	mmu-miR-302b*	001639	mmu-miR-704	001712	U87
002557	mmu-miR-302c*	001641	mmu-miR-706	001727	Y1

Supplementary Table S7. MicroRNA assays used for targeted analysis.

Assay names, IDs and miRBase/NCBI information are given for the assays used to follow up the ten most strongly associated microRNAs from the TaqMan® MicroRNA Array analysis. Endogenous control small RNA assays used for this analysis are shown in italics.

miRBase ID	Assay Name	Assay ID	miRBase/NCBI Accession Number
mmu-miR-192-5p	hsa-miR-192	000491	MIMAT0000517
mmu-miR-203-3p	hsa-miR-203	000507	MIMAT0000236
mmu-miR-224-5p	mmu-miR-224	002553	MIMAT0000671
mmu-miR-297b-5p	mmu-miR-297b	001626	MIMAT0003480
mmu-miR-484	hsa-miR-484	001821	MIMAT0003127
mmu-mir-592-5p	mmu-mir-592	002017	MIMAT0003730
mmu-miR-664-3p	rno-miR-664	001323	MIMAT0012774
mmu-miR-687	mmu-miR-687	001674	MIMAT0003466
mmu-miR-708-5p	mmu-miR-708	002341	MIMAT0004828
rno-miR-327	rno-miR-327	001328	MIMAT0000561
N/A	*snoRNA202*	*001232*	*AF357327*
N/A	*U6 snRNA*	*001973*	*NR_004394*
N/A	*U87*	*001712*	*AF272707*

Supplementary Table S8. mRNA assays used for predicted target analysis.

Assay IDs and NCBI information are given for the assays used to follow up the genes predicted to be targeted by the candidate microRNAs as determined by the DIANA-miRPath analysis. Endogenous control assays used for this analysis are shown in italics.

Gene Symbol	Assay ID	NCBI Accession Number
Acvr2a	Mm01331097_m1	NM_007396.4
Dusp5	Mm01266106_m1	NM_001085390.1
Fgf7	Mm00433292_m1	NM_008008.4
Gabarapl1	Mm00457880_m1	NM_020590.4
Mmp9	Mm00442991_m1	NM_013599.3
Pten	Mm01212530_m1	NM_008960.2
Rps6ka3	Mm00455829_m1	NM_148945.2
Smad4	Mm01262405_m1	NM_008540.2
Zfhx3	Mm01240016_m1	NM_007496.2
Gusb	*Mm01197698_m1*	*NM_010368.1*
Idh3b	*Mm00504589_m1*	*NM_130884.4*
